# Mapping post-translational modifications across amyloid-β deposits in Alzheimer’s disease: A neuropathologic survey with in vitro functional analysis of nitration

**DOI:** 10.21203/rs.3.rs-10769225/v1

**Published:** 2026-10-18

**Authors:** Sarah Basile, Fleur Schipper, Stefania Zimbone, Sanne M.M. Vermorgen, Francesco Bellia, Cristina Parisi, Salvatore Sortino, Jochen Walter, Maria Laura Giuffrida, Annemiek J.M. Rozemuller, Jeroen J.M. Hoozemans, Agata Copani, Baayla D.C. Boon

## Abstract

Background Amyloid-β (Aβ) pathology in Alzheimer’s disease (AD) includes distinct types of plaques and cerebral amyloid angiopathy (CAA). Post-translational modifications (PTMs) may define Aβ deposit-specific signatures. Among them, nitration of tyrosine-10 (3′-nitrotyrosine, 3-NT) links oxidative stress to Aβ modification and microglial activation, but its spatial distribution across deposit types and impact on aggregation dynamics remain unclear. Methods Parietal cortex tissue from AD and CAA cases (n = 19) and controls (n = 9) was analyzed by multiplex immunofluorescence for Aβ species (Aβaa1-8, AβN3pE, pSer8-Aβ), 3-NT, thioflavin-S, and CD68. Distinct deposits, including diffuse plaques (n = 70) classic cored plaques (n = 70), coarse-grained plaques (n = 40), capillary CAA (capCAA; n = 80), larger vessel CAA (n = 80), and leptomeningeal CAA (n = 25) were annotated, together with gray matter regions from control tissue lacking Aβ pathology (n = 90). In vitro assays evaluated how 3-NT affects Aβ aggregation kinetics, microglial morphology, and neuronal viability. Results We observed Aβ deposit-specific PTM signatures. Classic cored and coarse-grained plaques contained increased pyroglutamylated (Aβ _N3pE_ ) and phosphorylated (pSer8-Aβ) species compared to diffuse plaques. In contrast, 3-NT showed a distinct vascular predilection, with the highest relative burden in larger vessel and leptomeningeal CAA. 3-NT colocalized with β-sheet–rich thioflavin-S-positive fibrils and areas of CD68-positive microglia. In vitro, nitration slowed oligomer-to-fibril conversion but accelerated the growth of pre-formed fibrils and sustained microglial activation without increasing neuronal toxicity. Conclusions Distinct PTMs delineate the compartment-specific architectural signatures of Aβ deposits. Whereas AβN3pE and pSer8-Aβ broadly label parenchymal plaques, 3-NT shows a vascular predilection, particularly in leptomeningeal CAA. This compartmentalized pattern identifies nitration as a candidate marker of vascular Aβ pathology and a possible risk indicator for amyloid-related imaging abnormalities (ARIA) during anti-Aβ immunotherapy.

